# Opioid agonist treatment initiation and linkage for hospitalized patients seen by a substance use disorder consultation service

**DOI:** 10.1016/j.dadr.2022.100031

**Published:** 2022-02-07

**Authors:** Courtney D. Nordeck, Christopher Welsh, Robert P. Schwartz, Shannon Gwin Mitchell, Kevin E. O'Grady, Jan Gryczynski

**Affiliations:** a Friends Research Institute, Inc., Baltimore, MD, United States; b Department of Mental Health, Johns Hopkins Bloomberg School of Public Health, Baltimore, MD, United States; c Department of Psychiatry, University of Maryland School of Medicine, Baltimore, MD, United States; d Department of Psychology, University of Maryland, College Park, MD, United States

**Keywords:** Opioid use disorder, Opioid agonist treatment, Buprenorphine, Methadone, Hospitalization, Substance use consultation

## Abstract

**Background:**

Facilitating opioid agonist treatment (OAT) for opioid use disorder (OUD) is an important role of hospital substance use disorder (SUD) consultation services. In the *NavSTAR* trial, hospital patients receiving SUD consultation who were randomly assigned to patient navigation services for 3 months post-discharge had fewer readmissions compared to usual care.

**Methods:**

This secondary analysis examined hospital-based OAT initiation (pre-randomization) and community-based OAT linkage (post-discharge) among NavSTAR trial participants with OUD (*N=*314). Associations between OAT initiation and linkage, and patient demographics, housing status, comorbid SUD diagnoses, recent substance use, and study condition were examined using multinomial and dichotomous logistic regression.

**Results:**

Overall, 57.6% initiated OAT during hospitalization (36.3% methadone, 21.3% buprenorphine). Compared to participants not initiating OAT, participants who received methadone were more likely to be female (Relative Risk Ratio [RRR]=2.05, 95% CI=1.11, 3.82, *p*=0.02), while participants who received buprenorphine were more likely to report homelessness (RRR=2.57, 95% CI=1.24, 5.32, *p*=0.01). Compared to participants initiating methadone, participants initiating buprenorphine were more likely to be non-White (RRR=3.89; 95% CI=1.55, 9.70; *p*=0.004) and to report prior buprenorphine treatment (RRR=2.57; 95% CI=1.27, 5.20; *p*=0.009). OAT linkage within 30-days post-discharge was associated with hospital-based buprenorphine initiation (Adjusted Odds Ratio [AOR]=3.86, 95% CI=1.73, 8.61, *p=*0.001) and patient navigation intervention (AOR=2.97, 95% CI=1.60, 5.52, *p*=0.001).

**Conclusions:**

OAT initiation differed by sex, race, and housing status. Hospital-based OAT initiation and patient navigation were independently associated with linkage to community-based OAT. Hospitalization is a reachable moment to begin OAT to alleviate withdrawal and facilitate treatment continuity post-discharge.

## Highlights

• Hospital-based opioid agonist treatment (OAT) differed by sex, race, and housing status.
• Hospital-based OAT was associated with linkage to community-based treatment.
• Hospitalization is a reachable moment to initiate OAT.

## 1 Introduction

The opioid use disorder (OUD) and overdose crises continue to be major public health problems in the United States. In 2020, there was over 93,000 estimated drug overdose deaths, with nearly 75% involving an opioid (Ahmad et al., 2021). Opioid agonist treatment (OAT) with methadone and buprenorphine is a safe, effective, and lifesaving treatment option associated with reduced risk of opioid overdose mortality (Sordo et al., 2017; Volkow et al., 2014; Wakeman et al., 2020). While the effectiveness is well-established, only a small proportion of individuals with OUD receive OAT due to numerous barriers, spanning financial, geographic, and attitudinal domains (Priester et al., 2016; Sharma et al., 2017). As a result, OAT is highly underutilized and contributes to a significant treatment gap in many communities (Abraham et al., 2020; Andraka-Christou, 2021; The Medicaid Outcomes Distributed Research Network (MODRN) et al., 2021; Volkow, 2018).

Opioid use and related harms such as intravenous use are associated with several health conditions that result in hospitalization, including endocarditis, cellulitis, and soft tissue infections (Ronan and Herzig, 2016; Wurcel et al., 2016). Moreover, patients with OUD are at increased risk of death from drug-related causes, including but not limited to overdose, following discharge (King et al., 2021b). Despite the high utilization of hospital services among individuals with OUD, hospital-based management of OUD and addiction-specific interventions remain underutilized (Herscher et al., 2020; Naeger et al., 2016; Priest et al., 2020; Rosenthal et al., 2016). Initiating OAT in the hospital is feasible and has been found to facilitate linkage to outpatient treatment and reduce subsequent utilization of hospital services (Englander et al., 2019; Liebschutz et al., 2014; Shanahan et al., 2010; Wakeman et al., 2017). Thus, hospitalization can be a “reachable moment” during which OUD can be identified and OAT can be initiated if medically appropriate (Englander et al., 2017; Velez et al., 2017).

Hospital providers are equipped to assess for OUD and initiate treatment. Unfortunately, providers may be unaware that they are able to initiate OAT in the hospital or may feel uncomfortable doing so due to limited training (Wakeman et al., 2016). In recognition of this care gap, some hospitals have established specialized substance use disorder (SUD) consultation services that provide informed recommendations to attending medical teams regarding withdrawal management, OAT initiation and continuation, or referrals to community-based treatment (Englander et al., 2017; McDuff et al., 1997; Nordeck et al., 2018; Priest and McCarty, 2019; Trowbridge et al., 2017; Wakeman et al., 2017; Weinstein et al., 2018). Given that methadone and buprenorphine are protective against fatal overdose, initiating OAT in the hospital, and subsequently facilitating continuity of such care post-discharge, is a particularly important function of the hospital-based SUD consultation team (Herscher et al., 2020; Sordo et al., 2017; Wakeman et al., 2020). Furthermore, SUD consultation services have the potential to increase trust between patients and providers and improve substance use related patient outcomes following discharge (King et al., 2021a, 2020).

The current study is a secondary analysis from the *Navigation to Avoid Rehospitalization (NavSTAR)* study, a randomized controlled trial (RCT) that examined the effectiveness of patient navigation services in reducing hospital readmissions. The NavSTAR study found that patient navigation services reduced inpatient admissions and emergency department visits through 12-month follow-up. To build upon the evidence base for hospital-based initiation of OAT and OUD treatment linkage post-discharge, the current study examined participant characteristics, hospital-based OAT initiation, and post-discharge treatment linkage and retention among a subgroup of NavSTAR trial participants with OUD. Specifically, the aims of the current study were to examine the association between participant and service characteristics and (1) initiation of OAT in the hospital (any initiation and by medication type) [occurring prior to study enrollment/randomization], and (2) linkage to community-based OAT [post-discharge].

## 2 Methods

The current study is a secondary subgroup analysis of data collected in the NavSTAR study, which compared a patient navigation intervention to usual care among 400 medical or surgical inpatients with comorbid alcohol, cocaine, and/or opioid use disorder. All participants in the study received services from and were referred to the study by a hospital-based SUD consultation service. Detailed methods and outcomes are reported elsewhere (Gryczynski et al., 2021; Nordeck et al., 2020). The study was approved by the University of Maryland Institutional Review Board. All participants provided written informed consent.

### 2.1 Setting and service context

The NavSTAR study was conducted at the University of Maryland Medical Center (UMMC), a large academic hospital in Baltimore, MD. UMMC operates a SUD consultation service that has been in continuous operation for several decades (McDuff et al., 1997). The service is consulted by attending hospital teams when it is suspected that a patient may have a substance use problem that requires treatment, might be associated with or is causing health problems, and/or might interfere with adherence to post-discharge recommendations. The service is comprised of a multidisciplinary team, which, at the time of the study, included one full-time equivalent addiction boarded psychiatrist (divided between three attendings), a licensed addiction counselor, a licensed social worker, two nurses, and a rotating team of medical and psychiatric residents and addiction psychiatry/medicine fellows. The team conducts bedside assessments, provides motivational counseling regarding substance use, and makes recommendations regarding withdrawal management, OAT initiation or continuation, and community-based referrals. The team is well-established and widely utilized within the hospital (servicing approximately 2,000 high-risk patients annually). Participants randomized to usual care received these routine services from the SUD consultation team with no proactive follow-up from the team following discharge.

Participants randomized to the NavSTAR patient navigation condition received usual care from the SUD consultation service, as described above, in addition to patient navigation services delivered by Masters-level patient navigators. A navigator met with patients at the bedside to conduct an initial assessment of needs and barriers to care and develop a post-discharge plan. The patient navigator provided proactive care coordination services for up to three months post-discharge. These services aimed to link participants to available resources in the community for SUD treatment, recommended medical care, and basic needs. Patient navigators worked to resolve identified internal and external barriers (e.g., ambivalence about treatment, coordinating transportation, assisting with paperwork/scheduling, accompanying participants to appointments). Navigators were also able to access a modest support fund to cover costs as needed (e.g., medication co-payments, transportation).

### 2.2 Participants

All participants were patients hospitalized between March 2015 and May 2018 who received a SUD consultation. Participants were recruited from various hospital services including Internal Medicine (and various subspecialties), Trauma, Neurology and Surgery. For study inclusion, patients were required to: 1) be 18 or older; 2) have Baltimore City residency (or homeless); 3) meet DSM-5 criteria for alcohol, cocaine, and/or opioid use disorder; and 4) be willing and able to provide informed consent. Patients were excluded if they: 1) were enrolled in SUD treatment at the time of the index hospitalization; 2) were pregnant or in the hospital's Labor and Delivery unit; 3) had a planned discharge to hospice or long-term inpatient care (discharges to acute or sub-acute skilled nursing facilities were eligible); or 4) were hospitalized for a suicide attempt. Patients who received a consultation and were provided routine services from the SUD consultation service were referred to research staff for eligibility screening. After determining eligibility and obtaining informed consent, research staff completed a baseline interview at the bedside. Following the baseline interview, participants were randomly assigned to one of two study conditions: (1) usual care or (2) the NavSTAR patient navigation intervention.

For the current study, the analytic sample consists of participants in both study conditions who met DSM-5 criteria for OUD at baseline (*n*=314; 78.5% of the entire sample).

### 2.3 Data collection

Data were collected on sociodemographic characteristics, substance use behavior and history, and other risk behaviors. Research staff also abstracted information about the index hospitalization using the electronic health record (EHR), including admission and discharge diagnoses, housing status, and opioid pain medication.

#### 2.3.1 Hospital-based OAT initiation

Research staff abstracted data from SUD consultation records on OAT initiation with methadone and buprenorphine, which occurred prior to study enrollment and randomization. We defined OAT initiation as cases where (1) the SUD consultation made a recommendation for OAT to the medical team and (2) it was confirmed that participants received OAT medication during their hospitalization. Of note, hospital-based methadone and buprenorphine doses were adjusted based on a patient's symptoms and other clinical considerations. Further, hospital-based OAT initiation occurred prior to the delivery of the NavSTAR intervention.

#### 2.3.2 Community-based treatment linkage

Self-reported data on community-based treatment entry were collected during follow-up interviews conducted at 3-, 6-, and 12-months post-discharge. Specifically, participants recounted details of any community-based treatment episodes after discharge, including information on treatment entry dates, type of treatment setting, whether medication was provided, and whether the participant was still engaged in treatment at the time of the interview.

We defined community-based OAT linkage if the participant (1) reported a treatment start date within 30 days of discharge from the index hospitalization and (2) reported receiving buprenorphine or methadone for OUD as part of that treatment episode. We classified 30-day treatment retention using self-reported data on the number of days the participant engaged in community-based OAT following linkage to a community-based program post-discharge.

At the time of this study, there was no established stand-alone bridge clinic for post-discharge treatment. However, the hospital system operates an opioid treatment program (OTP) that provides methadone and buprenorphine, as well as another non-OTP program that offers buprenorphine. These programs are within several blocks of the hospital and provide next-day appointments for patients referred from the SUD consultation service. Patients discharged on Friday or Saturday (and unable to be admitted to the OTP until Monday) were seen in the hospital's psychiatric emergency department where they were administered methadone. The consultation service also has established relationships with other outpatient programs (OTP and non-OTP) in other areas of the city which will see patients the next day for methadone. Prescriptions for buprenorphine are provided to patients to bridge them to their intake if not next day. The hospital system does not have the capacity to provide direct follow-up for hospital patients once discharged, though patients with direct referrals from the SUD consultation service can be prioritized for admission into the system's community-based programs.

### 2.4 Statistical analysis

We used descriptive statistics to examine OAT initiation across participant characteristics. We used a multivariable, multinomial logistic regression model to compare participants not initiated on OAT to participants initiated on either methadone or buprenorphine, respectively, using sex, race, age, homelessness, co-morbid SUD diagnoses (cocaine or alcohol), days of illicit opioid use, injection drug use, and previous treatment history as explanatory variables. The focus of the multinomial logistic regression was to characterize differences between participants initiated on either medication type compared to participants who did not receive medication, as well as differences between participants initiated on buprenorphine compared to participants initiated on methadone. In addition to considering participant characteristics, we described clinical aspects that could influence OAT initiation, such as pain management with opioid analgesics and medical diagnoses.

We used a multivariable logistic regression model to examine linkage to community-based OAT within 30 days of discharge, using the above explanatory variables, as well as hospital-based OAT initiation (methadone, buprenorphine, reference=neither) and study condition (patient navigation, reference=usual care). Study intervention condition was included as a predictor because linkage to community-based OAT post-discharge may have been related to the intervention, as facilitating linkage to SUD treatment was a core function of the patient navigators. Furthermore, we extended the model to consider the interaction between hospital-based OAT initiation and study condition. Finally, we used descriptive statistics to examine rates of 30-day retention in community-based OAT. All statistical analyses were conducted in Stata Version 16.

## 3 Results

### 3.1 Participant characteristics

Of the 314 participants with OUD, most (92.7%) met criteria for severe OUD (≥ 6 diagnostic symptoms) and reported opioid withdrawal symptoms in the past 30 days (>95%). Descriptive statistics of the analytic sample are shown in Table 1. The sample was 54.8% male, with a mean age of 44.1 years (*SD*=12.3). Over half of the sample (60.2%) had co-morbid cocaine use disorder, while 21.7% had co-morbid alcohol use disorder. Nearly two-thirds (64.3%) reported recent injection drug use and 46.8% reported experiencing homelessness at baseline. Over half of the sample (*n*=181, 57.6%) were initiated on OAT, with 36.3% initiated on methadone and 21.3% initiated on buprenorphine (occurring prior to study enrollment/randomization). Approximately half of the sample were randomized to receive the patient navigation intervention post-discharge (*n*=163; 51.9%).

**Table 1:** Patient characteristics by initiation of opioid agonist treatment (OAT).

	No OAT n = 133	Methadone n = 114	Buprenorphine n = 67	Total n = 314
Sex				
Male – n (%)	84 (63.2)	50 (43.9)	38 (56.7)	172 (54.8)
Female – n (%)	49 (36.8)	64 (56.1)	29 (43.3)	142 (45.2)
Age (years) – mean (SD)	45.9 (12.7)	40.2 (10.7)	47.3 (12.6)	44.1 (12.3)
Race				
White – n (%)	54 (40.6)	80 (70.2)	17 (25.4)	151 (48.1)
Non-White – n (%)	79 (59.4)	34 (29.8)	50 (74.6)	163 (51.9)
Substance use disorder criteria				
Cocaine use disorder – n (%)	74 (55.6)	82 (71.9)	33 (49.3)	189 (60.2)
Alcohol use disorder – n (%)	34 (25.6)	16 (14.0)	18 (26.9)	68 (21.7)
Drug use behavior – mean (SD)				
Days of heroin/street opioid use (past 30)	20.6 (11.0)	27.7 (5.7)	25.9 (7.8)	24.3 (9.3)
Days of NMPO use (past 30)	3.2 (7.5)	3.2 (7.8)	0.9 (3.0)	2.7 (7.0)
Injection drug use – n (%)	77 (57.9)	94 (82.5)	31 (46.3)	202 (64.3)
Previous methadone treatment – n (%)	68 (51.1)	79 (69.3)	35 (52.2)	182 (58.0)
Previous buprenorphine treatment – n (%)	60 (45.1)	42 (36.8)	38 (56.7)	140 (44.6)
Homelessness – n (%)	51 (38.4)	64 (56.1)	32 (47.8)	147 (46.8)
Linked to OAT within 30 days of discharge*	24 (21.6)	29 (32.2)	26 (52.0)	79 (31.5)

⁎ Treatment linkage data is limited to participants who completed at least 1 follow-up assessment (n=251; 111 No OAT, 90 Methadone, 50 Buprenorphine).

### 3.2 Multinomial logistic regression for OAT initiation type (methadone vs buprenorphine vs no initiation)

Results of the multinomial logistical regression are reported in Tables 2 and 3. Compared to participants not initiated on OAT, participants initiated on methadone were more likely to be female (Relative Risk Ratio [*RRR*]=2.05, *95% CI*=1.11, 3.82, *p*=0.02), and to report greater days of heroin/illicit street opioid use (*RRR*=1.12, *95% CI*=1.07, 1.17, *p*<0.001) and non-medical prescription opioid (NMPO) use (*RRR*=1.06, *95% CI*=1.01, 1.12, *p*=0.02) in the 30 days prior to the index hospitalization. There were no other significant differences between the group initiated on methadone (compared to the group not initiated) for the remaining explanatory variables.

**Table 2:** Relative risk ratios of OAT initiation, by medication type (n = 314). *Note:* Reference group is no OAT. RRR = Relative Risk Ratio, 95% CI = 95% Confidence Interval, NMPO = non-medical prescription opioid.

	Methadone		Buprenorphine	
	Adjusted RRR (95% CI)	p-value	Adjusted RRR (95% CI)	p-value
Sex (ref=Male)				
Female	**2.05 (1.11, 3.82)**	**0.02**	1.58 (0.81, 3.08)	0.18
Age (years)	0.99 (0.96, 1.02)	0.54	1.00 (0.97, 1.03)	0.95
Race (ref=White)				
Non-White	0.62 (0.29, 1.32)	0.22	**2.40 (1.01, 5.70)**	**0.046**
SUD criteria (ref=no disorder)				
Cocaine use disorder	1.21 (0.63, 2.33)	0.56	0.60 (0.30, 1.17)	0.13
Alcohol use disorder	0.70 (0.32, 1.53)	0.37	1.07 (0.50, 2.27)	0.87
Drug use behavior				
Days of heroin/street opioid use (past 30)	**1.12 (1.07, 1.17)**	**<0.001**	**1.06 (1.02, 1.10)**	**0.004**
Days of NMPO use (past 30)	**1.06 (1.01, 1.12)**	**0.02**	0.94 (0.86, 1.02)	0.13
Injection drug use (ref=no)	1.07 (0.48, 2.41)	0.87	0.59 (0.27, 1.30)	0.19
Previous methadone treatment (ref=no)	1.64 (0.88, 3.04)	0.12	1.11 (0.58, 2.14)	0.76
Previous buprenorphine treatment (ref=no)	0.61 (0.34, 1.11)	0.10	1.57 (0.82, 3.01)	0.17
Homelessness (ref=no)	1.76 (0.91, 3.39)	0.09	**2.57 (1.24, 5.32)**	**0.01**

**Table 3:** Relative risk ratios of buprenorphine initiation compared to methadone (n=314).

	Buprenorphine	
	Adjusted RRR (95% CI)	p-value
Sex (ref = Male)		
Female	0.77 (0.37, 1.57)	0.47
Age (years)	1.01 (0.98, 1.05)	0.57
Race (ref = White)		
Non-White	**3.89 (1.55, 9.70)**	**0.004**
SUD criteria (ref = no disorder)		
Cocaine use disorder	0.49 (0.23, 1.04)	0.06
Alcohol use disorder	1.53 (0.64, 3.68)	0.34
Drug use behavior		
Days of heroin/street opioid use (past 30)	0.95 (0.90, 1.00)	0.06
Days of NMPO use (past 30)	**0.88 (0.80, 0.96)**	**0.006**
Injection drug use (ref= no)	0.55 (0.22, 1.35)	0.19
Previous methadone treatment (ref= no)	0.68 (0.33, 1.40)	0.30
Previous buprenorphine treatment (ref= no)	**2.57 (1.27, 5.20)**	**0.009**
Homelessness (ref= no)	1.46 (0.67, 3.19)	0.34

*Note:* Reference group is methadone initiation. RRR=Relative Risk Ratio, 95% CI=95% Confidence Interval, NMPO=Non-medical prescription opioid.

Compared to participants not initiated on OAT, participants initiated on buprenorphine were more likely to be non-White (*RRR*=2.40, *95% CI*=1.01, 5.70, *p*=0.046), to report experiencing homelessness (*RRR*=2.57, *95% CI*=1.24, 5.32, *p*=0.01), and to report greater days of heroin/illicit street opioid use in the 30 days prior to the index hospitalization (*RRR*=1.06, *95% CI*=1.02, 1.10, *p*=0.004).

Compared to participants who initiated methadone, participants who initiated buprenorphine were more likely to be non-White (*RRR*=3.89; *95% CI*=1.55, 9.70; *p*=0.004) and to report previous buprenorphine treatment experience (*RRR*=2.57, *95% CI*=1.27, 5.20, *p*=0.009). Compared to participants who initiated methadone, participants initiated on buprenorphine reported fewer days of NMPO use (*RRR*=0.88, *95% CI*=0.80, 0.96, *p*=0.006).

### 3.3 Other potential contributors to OAT initiation

We considered several other factors that could affect OAT initiation, including pain management, discharge diagnosis, patient interest, and benzodiazepine misuse. Among individuals not initiated on OAT (*n*=133), prescription opioids for pain (e.g., hydromorphone, morphine) were noted by the SUD consultation team for 77.4%, compared to 18.2% for participants who did initiate OAT (*p<*0.001). Of participants on opioid analgesics, 76% were not initiated on OAT, 21% initiated methadone, and <3% initiated buprenorphine (*p<*0.001).

Two of the authors (CW and RPS) reviewed participants’ discharge diagnoses, identifying several medical conditions that could influence OAT initiation, or choice of medication: cardiac issues/QTc prolongation (*n*=8), hepatic issues (*n*=8), ongoing pain (*n*=5), and other medical complications (*n*=3). However, actual OAT initiation patterns did not differ from the larger sample for these cases.

Among individuals who did not initiate OAT, five participants (3.8%) did not express interest in OAT during hospitalization. Three other participants (2.3%) were recommended for OAT during their admission but left against medical advice prior to receiving medication.

Past 30-day non-medical use of sedatives (including benzodiazepines) was reported by 14% of the sample, but at low frequency on average (≤3/30 days). While not included in the main analyses due to small numbers, additional exploratory analyses found that sedative misuse was not associated with either any OAT initiation, or medication type.

### 3.4 Logistic regression of community OAT linkage post-discharge and rates of 30-day retention

Approximately 80% of the sample completed at least one follow-up interview following the index hospitalization (*n*=251) and provided data on community-based OAT.

Results of the logistic regression examining linkage to community-based OAT within 30-days post-discharge are presented in Table 4. Participants were significantly more likely to link to community-based OAT within 30-days post-discharge if they had received buprenorphine during the index hospitalization (Adjusted Odds Ratio [*AOR*]=3.86, *95% CI*=1.73, 8.61, *p*=0.001) and if they were randomized to the patient navigation intervention condition (*AOR*=2.97, *95% CI*=1.60, 5.52, *p*=0.001). Participants with co-morbid alcohol use disorder also were more likely to link with community-based OAT within 30 days of discharge (*AOR*=2.49, *95% CI*=1.21, 5.15, *p*=0.01). Compared to no medication, there was no significant association between methadone initiation during the index hospitalization and linkage to community-based OAT (*AOR*=1.72, *95% CI*=0.80, 3.69, *p*=0.17). However, there was a significant association of any medication initiation (either buprenorphine or methadone) with community-based OAT linkage (joint 2-degree-of-freedom contrast compared to no medication: χ^2^(2) =10.85, *p*=0.004). In changing the reference category to examine initiation with buprenorphine versus methadone, this comparison did not reach statistical significance (*p*=0.06).

**Table 4:** Odds ratios of community-based OAT linkage within 30 days of hospital discharge (n = 251).

	Adjusted OR (95% CI)	p-value
Intervention condition (ref=Usual Care)		
Patient Navigation Intervention	**2.97 (1.60, 5.52)**	**0.001**
OAT initiation in hospital (ref=Neither)		
Initiated MTD during hospitalization	1.72 (0.80, 3.69)	0.17
Initiated BUP during hospitalization	**3.86 (1.73, 8.61)**	**0.001**
*Joint test of any initiation (MTD or BUP)*	*(χ^2^(2)) =* 10.34	**0.004**
Sex (ref=Male)		
Female	1.05 (0.56, 1.97)	0.87
Age (years)	0.99 (0.96, 1.02)	0.35
Race (ref=White)		
Non-White	1.10 (0.50, 2.42)	0.82
SUD criteria (ref=no disorder)		
Cocaine use disorder	0.71 (0.37, 1.39)	0.32
Alcohol use disorder	**2.49 (1.21, 5.15)**	**0.01**
Drug use behavior		
Days of heroin/street opioid use (past 30)	1.02 (0.98, 1.06)	0.39
Days of NMPO use (past 30)	1.00 (0.95, 1.05)	0.90
Injection drug use (ref=no)	1.43 (0.62, 3.27)	0.40
Previous methadone treatment (ref=no)	1.41 (0.73, 2.70)	0.30
Previous buprenorphine treatment (ref=no)	1.37 (0.75, 2.51)	0.31
Homelessness (ref= no)	0.64 (0.33, 1.25)	0.19

*Note:* Reference group for the dependent variable is No OAT within 30 days of hospital discharge. OR = Odds Ratio, 95% CI = 95% Confidence Interval, MTD = Methadone, BUP = Buprenorphine, NMPO = Non-medical prescription opioid.

An extension of the model testing an interaction effect between hospital OAT initiation and intervention condition found the interaction to be non-significant [2-degree-of-freedom contrast: χ^2^(2) =1.21, *p*=0.55], indicating that the patient navigation intervention was not differentially effective in facilitating treatment linkage based on whether the participant started OAT in the hospital. Rather, both in-hospital OAT initiation and patient navigation services post-discharge were independently associated with linkage to community-based OAT.

Among the subsample of participants who reported linkage to OAT (*n*=79), 88.6% were engaged for at least 30-days (*n*=70). Bivariate comparisons using χ^2^ tests showed no statistically significant differences in rates of 30-day retention based on receipt of hospital-based OAT (83.3% no OAT vs. 86.2% methadone vs. 96.2% buprenorphine) or the patient navigation intervention (90.9% NavSTAR *vs*. 83.3% usual care).

## 4 Discussion

The current overdose epidemic in the US highlights the importance of identifying individuals with OUD and engaging them in effective treatment. Hospitalization offers a critical moment during which clinicians can safely initiate treatment to alleviate withdrawal symptoms, encourage continued engagement post-discharge, reduce opioid-related harms, and increase the likelihood of adhering to recommended medical and substance use treatments.

In this study, most patients met criteria for severe OUD (i.e., ≥6 DSM-5 symptoms). Additionally, this sample reported high rates of injection drug use, frequent drug use days, and homelessness. Thus, this sample represents vulnerable, high-risk patients who could benefit from hospital-based OAT initiation, as such care could reduce risks related to injection drug use or infectious disease and promote better quality of life (MacArthur et al., 2012; Ponizovsky and Grinshpoon, 2007; Woody et al., 2014). Examining differences between patients who do and do not initiate hospital-based OAT can identify potential service disparities and address related treatment gaps. Moreover, examining associations between hospital-based OAT initiation and subsequent linkage to community-based OAT can help to promote strategies and policies that may reduce gaps in continuity of care.

Within this sample, participants who were initiated on buprenorphine during hospitalization were nearly four times more likely to enter community-based OAT within 30 days of discharge compared to participants not initiated on OAT. This is consistent with other studies that have found that OAT initiation in hospital settings is associated with increased engagement in outpatient care (D'Onofrio et al., 2017; Liebschutz et al., 2014; Regan et al., 2021) and highlights the efficacy of buprenorphine initiation during hospitalization. While methadone was not independently associated with increased linkage to treatment in the community, there was an overall effect of OAT initiation on community-based treatment linkage, and hospital providers should consider factors such as available community treatment options, patient preference, and medical comorbidities when initiating OAT. Although prior studies have found that retention in methadone is often better than in buprenorphine (Mattick et al., 2014), there may be fewer barriers to treatment linkage and continuity after discharge with buprenorphine than with methadone. For example, community-based methadone programs may have more onerous intake processes and often require new patients to attend the program daily for medication dispensing, whereas buprenorphine may offer fewer barriers to continuity across settings.

The study's patient navigation intervention was also independently associated with increased odds of community-based treatment engagement, when controlling for other factors. The patient navigation intervention primarily focused on SUD treatment linkage; however, navigators routinely resolved challenges ranging from acute medical needs to transportation barriers that may have increased participant readiness to engage in SUD treatment once these problems were addressed. Given the competing needs of patients during acute hospitalizations, hospital systems should consider utilizing addiction-focused specialists in discharge planning and transitional care to promote this continuity. There is a high need for targeted interventions for hospitalized patients with SUDs, such as patient-centered case management services, that can facilitate linkage to community-based treatment upon discharge and provide motivational and logistic support for individuals who are newly engaged in treatment and at risk of early treatment discontinuation.

In this sample, co-morbid alcohol use disorder was associated with increased likelihood of community-based OAT linkage, which is in contrast with other studies that have found associations between alcohol use disorder diagnosis and treatment non-adherence in general populations (Macmadu et al., 2021; Nolan et al., 2016; Soyka, 2015). Researchers hypothesize that other substance use and mental health-related diagnoses may reduce the ability to engage in treatment (Macmadu et al., 2021). However, in a setting that emphasizes the relationship between these comorbidities (e.g.., through connection with SUD consultation teams), individuals with dual diagnoses may benefit from focused logistical support and motivation to connect with outpatient care.

We also identified sociodemographic differences in the hospital-based OAT initiation. First, we found that that women were more likely to be initiated on methadone, a contrast to extant research showing that women are likely to experience multiple barriers to treatment access and are less likely to seek care (McHugh et al., 2018). Hospitalization may serve as an opportunity to resolve various barriers to treatment-seeking experienced by women, such as fear of stigma or denial (Taylor, 2010).

Second, compared to participants who received methadone, participants who received buprenorphine during hospitalization were substantially more likely to be non-White. This contrasts with research that has found lower access to buprenorphine treatment in non-White individuals. Several factors are associated with decreased access to treatment among racial and ethnic minority groups, including clinician attitudes and bias and policies that limit accessibility (Alegria et al., 2011). As noted above, this finding suggests that hospitalization can be a reachable moment for populations that are otherwise underserved in traditional treatment settings. Further, this study was conducted in Baltimore, which has comparatively robust availability of publicly funded buprenorphine and methadone treatment. It is possible that the dynamics of treatment availability in this local context may alter the usual patterns of service disparities that are seen along lines of race, sex, and other patient characteristics. Relatedly, prior history of buprenorphine treatment increased the odds of hospital-based buprenorphine initiation (compared to methadone initiation), indicating that factors such as patient experience and preference may influence treatment decisions. The context of the SUD consultation service is also important to consider, as this team is staffed by a group of clinicians in addiction medicine that are experienced in the field and well-accustomed to using medications to treat OUD.

Finally, similar to other studies of OAT initiation in hospital settings, this study found that experiencing homelessness was positively associated with OAT initiation, specifically buprenorphine (Englander et al., 2020). This could be indicative that these individuals may not be accessing treatment outside of hospital settings and highlights the importance of considering such social factors when engaging vulnerable populations in care and developing treatment plans.

While this study identified certain patient characteristics that were associated with OAT initiation and which medication people started, the overarching finding of greatest importance is that most participants were able to successfully initiate OAT with either buprenorphine or methadone during a medical hospitalization. Among participants who did not initiate OAT, most were receiving opioids for analgesia (which may have temporarily resolved acute withdrawal). Opioid analgesics (whether prescribed prior to or during hospitalization) can complicate the logistics of OAT initiation with respect to choice of medication, dosage, and frequency. This sample was a medically complex, socially vulnerable one characterized by a wide range of presenting medical problems, for which multiple comorbidities were common. Our findings highlight the important role of SUD consultation services in facilitating OAT in the hospital setting.

## 5 Limitations

This study has several limitations. First, we were likely unable to account for all variables that influence OAT initiation during hospitalization or linkage post-discharge, and many that we considered were measured imperfectly. While we obtained information on the important issues of opioid analgesia (which may be a complicating factor to initiating OAT) and discharge diagnoses, our data abstraction did not capture other factors that could affect treatment decision-making and discharge planning, such as nuances of patients’ medical conditions or specific practices surrounding initiation of opioid agonist medications such as dose and timing. Moreover, because recruitment occurred from 2016-2018, the study was conducted during the early transition to fentanyl in the illicit opioid supply. There is emerging evidence of precipitated withdrawal among patients using fentanyl when starting buprenorphine treatment (Varshneya et al., 2021). Thus, it is important for providers to know which opioids patients report using and to proceed with medication initiation cautiously in this respect.

Second, the study was conducted in a large, urban teaching hospital with a well-established SUD consultation service, which may limit generalizability of these findings for hospitals without such specialized capacity, or hospitals with less mature or under-resourced SUD consultation services.

Third, outcome data on linkage to and retention in community-based OAT was self-reported by participants and may be subject to error or bias, for example, due to recall error or social desirability.

Finally, the sample of patients who enrolled in the NavSTAR trial exhibited high rates of severe OUD, which may not generalize to populations with less severe OUD. While a comprehensive accounting of the clinical decision process for OAT initiation is beyond the scope of this study, such treatment decisions are likely a complex interplay of patient characteristics, patient/provider preferences, underlying medical conditions (e.g., cardiac or hepatic issues that may favor buprenorphine, ongoing pain issues that may favor methadone), other treatments received during the hospitalization (especially opioid analgesics), and prospects for OAT continuity upon discharge.

## 6 Conclusion

This study highlights that many hospitalized patients who meet diagnostic criteria for OUD are appropriate for OAT and can be initiated on such treatment during hospitalization. Moreover, initiating OAT in the hospital, as well as providing patient navigation services post-discharge, are independently associated with considerably higher rates of linkage to OAT in the community within 30-days post-discharge. Hospitalization offers a critical opportunity to expand access to OAT for patients with comorbid OUD to alleviate withdrawal symptoms and to facilitate continuity of care after discharge. Specialty SUD consultation services are poised to play an important role in helping to address the opioid crisis. Inpatient care for hospitalized patients with OUD can be complex, thus it is important to identify patterns and disparities in treatment delivery in order to optimize care for high-risk populations.

## Disclosures

Unrelated to the present study, JG is part owner of COG Analytics and has received research funding (paid to his institution and including project-related salary support) from Indivior. He is also Principal Investigator of a NIDA-funded study that is receiving medication in kind from Alkermes and Indivior. RPS has consulted with Verily Life Sciences.

## Contributors

CD Nordeck managed the data collection for the study, conducted literature review and statistical analysis for this manuscript, and drafted the manuscript. J Gryczynski acted as the Principal Investigator for the *NavSTAR* study, designed the study protocol, assisted with the data analyses and contributed to the writing of the manuscript. C Welsh was the director of the addiction consultation service and contributed to the writing of the manuscript. KE O'Grady provided guidance on the data analyses. RP Schwartz and SG Mitchell contributed to the study design and manuscript preparation. All authors contributed to the interpretation of the findings and critically reviewed the final manuscript. All authors approved the final manuscript.

## Declaration of Competing Interest

None related to the presented research.

Unrelated to the present study, J Gryczynski is part owner of COG Analytics and has received research funding (paid to his institution and including project-related salary support) from Indivior. J Gryczynski and RP Schwartz are involved in a separate NIDA-funded study that is receiving free study medication from Alkermes and Indivior (unrelated to the present study). RP Schwartz has consulted with Verily Life Sciences. The remaining authors report no conflicts of interest.
